# Matrix metalloproteinases in autism spectrum disorders

**DOI:** 10.1186/2049-9256-1-16

**Published:** 2013-09-17

**Authors:** Morsi W Abdallah, Tanja M Michel

**Affiliations:** Department of Psychiatry and Psychotherapy, Rostock University Medical Center, Gehlsheimer Straße 20, Rostock, 18147 Germany; Department of Child and Adolescent Neuropsychiatry, Rostock University Medical Center, Gehlsheimer Str. 20, Rostock, 18147 Germany

**Keywords:** Fragile-X syndrome, Gelatinase, Hyperplasticity, Inflammation, Neurodevelopmental disorders, Protease

## Abstract

Autism Spectrum Disorders (ASD) are group of developmental disabilities with a complex neurobiological basis including putative changes in the immune system. They are characterized by pervasive qualitative abnormalities in social interactions, communication, and stereotyped behaviour. Matrix metalloproteinases (MMPs) represent a group of proteases which play an important role in neuroinflammation and neurodevelopment. Therefore, they possibly have a crucial function in the etiopathology of ASD. In this review, we summarize the plausibility of the hypothesis that MMPs are involved in the neuropathology of ASD. Possible pathways through which MMPs can contribute to the pathogenesis of ASD are discussed including neuroinflammatory mechanisms inclusive of mediating neuropathological effects of infections, the associations between MMPs and other biomarkers such as cytokines, chemokines and neurotrophic factors. Despite sufficient evidence for such an involvement of MMPs in the neuropathology of ASD, they have not yet been extensively studied in this context. Thus, further research in this field is not only urgently needed but also very promising and may also lead to new therapeutic approaches.

## Review

### Introduction

Matrix metalloproteinases (MMPs), discovered back in 1962, are a family of at least 28 endopeptidases. They encompass a large family of proteases and share many similarities in their structure, regulation and function [1]. In their active form, MMPs play a number of important roles not only in physiological conditions but also in pathological states [2]. They are essential for various physiological processes such as embryonic development, morphogenesis and remodelling. Furthermore, they have been implicated in a number of key pathologic processes including inflammation, fibrosis, arthritis and cancer [1].

Additionally, MMPs play a crucial role in the development of the central nervous system (CNS) and neurogenesis as well as during phases of neuroinflammation [2, 3], a frequently reported finding in children with Autism Spectrum Disorders (ASD) [4].

Autism spectrum disorders (ASD), or pervasive developmental disorders (PDD), as termed in the International Classification of Diseases, 10^th^ version [5], refer to a group of heterogeneous neurodevelopmental disorders characterized by qualitative impairments in social interaction, communication and repetitive stereotypic behaviour [6, 7]. While accumulating evidence suggests that immune processes play a key role in the pathophysiology of ASD [8], no definitive biologic screening or diagnostic tools have been universally accepted, and the diagnostic standards are still based on behavioural criteria [6].

This review first introduces briefly members of the MMPs and their biochemistry. This is followed by a short description of their physiological functions within CNS as well as their involvement in pathological states. The review focuses mainly on the potential pathways through which MMP’s can contribute to the etiopathology of ASD.

### The structure and biochemistry of MMPs

MMPs along with the ADAMs (A Disintegrin And Metalloproteinase) and the ADAMTs (A Disintegrin And Metalloproteinase with Thrombospondin Motifs) are subgroups of the larger metzincin superfamily [9] that are collectively able to process and degrade various extracellular matrix (ECM) proteins. Based on their protein structure, MMPs are divided into four groups which include collagenases (such as MMP-1), stromelysins (MMP-3), gelatinases (MMP-2, MMP-7 and MMP-9), and membrane type metalloproteinases (MT-MMPs) such as (MMP-14) [2].

While MMPs were initially described to be the products macrophages and neutrophils acting on collagen, they are now known to be produced by different tissues and cell types, including osteoblasts, human umbilical vein endothelial cells, smooth muscle cells and keratinocytes [10]. They often bind with heparin sulphate glycosaminoglycans on the cell surface and have wide range of substrates [11].

The regulation of MMPs is complex starting at gene transcription, posttranslational activation of zymogens, and endogenous inhibition [12]. The synthesis and secretion of MMPs take place in inactive forms which are later activated by the loss of a 10-kDa propeptide either intracellularly or extracellularly. The activity of MMPs is balanced by the endogenous tissue inhibitors of metalloproteinases (TIMPs) and by α2-macroglobulin. The resulting equilibrium between production, activation, and inhibition prevents excessive proteolysis or inhibition [2, 10].

### MMPs in the CNS

MMPs play an important role in the development of the CNS as well as during pathological periods of inflammation and injury. Substrates of MMPs have important functions in normal CNS development during synaptogenesis, synaptic plasticity, and long-term potentiation (LTP) [13]. Several animal studies have documented the presence of different MMPs such as MMP-9 and MMP-2 in the brain [2, 13]. MMPs are mainly secreted by astrocytes and microglia within the CNS due to different triggers [14].

Alteration of MMPs expression has been detected in the nervous system in response to injury or neurological disease [15, 16]. MMPs have the ability to mediate the disruption of the blood brain barrier (BBB) by degrading the tight junctions’ proteins and basal lamina proteins, thereby leading to BBB leakage, leukocyte infiltration, brain edema, and hemorrhage. Furthermore, they regulate ECM protein destruction, remodelling and tissue inflammation in response to oxidative stress [17]. Additionally, several reports have illustrated MMPs involvement in wide range of neurological pathologies such meningitis [18], multiple sclerosis [19], Alzheimer’s disease [15], inflammatory myopathies [20] and tumors of the CNS such as glioma [21].

### Possible role of MMPs in the etiopathology of ASD

The neurobiological basis of ASD is complex, and several lines of research suggest that both genetic and envir-onmental factors contribute etiologically to ASD [22, 23]. Despite the extensive ongoing research, convergence towards a universal molecular pathway is still lacking, and ASD is still considered to have an idiopathic etiology in many individuals [22]. Although MMPs have been extensively investigated in several somatic and psychiatric disorders [10, 24], their role in the etiopathology of ASD have been less extensively examined. Most of the research in this field has been targeting MMPs levels in individuals with Fragile-X Syndrome (FXS) [25], a disorder where at least 30% of patients have features of ASD [26]. Several animal models of FXS have reported elevated levels of MMPs in the CNS [27]. This drove the main interest in this area was targeted toward Minocycline, an antibiotic which is widely used to treat acne and other skin infections but also inhibits matrix metalloproteinase (MMP)-9 [28]. Although the application of this drug to FXS patient in several clinical trials have revealed positive improvements in language, attention and behavioural improvements, research in this area is still ongoing [29].

Interestingly, in a recent study by Abdallah et al., [16], amniotic fluid samples for 331 ASD cases and 698 frequency-matched controls were analyzed for levels of MMP-9 along with other biomarkers utilizing a Danish historic birth cohort and Danish nationwide health registers Our results showed elevated levels of MMP-9 in ASD cases compared with controls and this was unrelated to FXS.

Contribution of MMPs to the etiopathology of ASD is biologically plausible through direct and indirect pathways which are not necessarily mutually exclusive. Several studies have reported disrupted synaptic pathways in some cases of ASD [30] along with abnormal formation of neuronal connections or elimination of inappropriate connections [31]. Besides, anomalies of brain structure have repeatedly been reported in cases with ASD [32].

MMPs play important roles in neuronal development and neuroplasticity [33]. They have also important functions in reactive synaptogenesis following brain injury [27]. Interestingly, several neuronal activity altered genes associated with ASD such as those encoding Neurexin (NRXN1) and Neuroligin (NLGN3) are processed by MMPs. Furthermore, elevated levels of MMPs can induce hyperplasticity state within the CNS that eventually leads to ASD, fitting well within the hyperreactivity/hyperplasticity model of the disease [34]. It is not clear though whether MMPs act directly or as an epiphenomenon to lead ultimately to ASD. This sheds the light on the potential role of MMPs in modulating neuroplasticity and neurogenesis indirectly through interaction with molecules such as neuroligins, integrins and growth factors [13].

MMPs can activate several neurotrophic factors (NFs), such as Brain-derived neurotrophic factor (BDNF), through cleavage processes of their proforms which can ultimately regulate neuronal survival, development, and synaptic plasticity [35].

Theoretically speaking, MMPs can also contribute to ASD pathology, on one hand, through inducing a neuroinflammatory state or through disruption of the BBB leaving the normally protected milieu of the CNS more vulnerable to the systemic circulation [36]. On the other hand, MMPs have also the ability to act through different inflammatory markers associated with ASD pathology through their proteolytic activity. Such markers include cytokines, chemokines, and reactive oxygen species (ROS) [37, 38].

MMPs have the ability to regulate various inflammatory processes at different levels including epithelial repair, defence mechanisms against microorganisms and through modulatory effects on cytokines and chemokines [39]. Furthermore, elevated levels of MMPs were reported in Experimental Allergic Encephalomyelitis (EAE), an animal model of a monophasic inflammatory demyelinating illness associated with ASD and other neuropsychiatric diseases. Interestingly, applying MMPs inhibitors has also showed antinflammatory effects [40]. Given that neuroimmune factors can directly affect brain development and that neuroinflammation can be a critical pathogenic factor in the development of ASD [4], MMPs can serve as important candidates to better understand the underlying neurodevelopmental pathology in ASD.

The triad of infections, ASD and MMPs is of special importance. Several studies have reported associations between infections and ASD. Such associations were proposed as early as 1963, with Dr. Krevelen reporting congenital rubella infection in a patient with infantile autism [41]. Both Congenital infections [42–44] and postnatal childhood infections [45, 46] were associated with high rate of ASD. On the other arm of the triad is the association between infections and MMPs. Elevated levels of MMPs (specifically MMP-1) were found associated with bacterial meningitis [18] and were correlated with cerebral injury and infection severity during infection. Also, there is an increased ROS-induced activity of MMPs during viral infections as a result of intracellular signalling by virion components or cytotoxic effects of viral non-structural proteins [47]. Probably, the direct contribution of infections to the rising ASD prevalence is limited [48]. However, their role triggering an inflammatory state is rather important [49]. Taken together, it is possible that MMPs can intermediate the neuropathological effects of infections (and probably other environmental insults) which eventually contribute to the development of ASD.

There is also growing evidence regarding the important role of cytokines and chemokines in mediating inflammatory effects on the neurodevelopmental trajectory in autism and other psychiatric disorders such as schizo-phrenia [50]. MMPs can regulate inflammatory response through their modulatory effects on cytokines and chemokines [39]. Accumulating evidence shows that MMPs can either promote or repress inflammation through proteolytic processing of inflammatory cytokines and chemokines [1]. For example, MMPs have the ability to amplify inflammatory response through activating cytokines such as Tumor necrosis factors α (TNF-α), Interleukin 1β (IL-1β) and wide range of chemokines. Elevated levels of TNF- α in amniotic fluid [51] cerebrospinal fluid [52], peripheral blood mononuclear cells [53, 54], whole blood samples [55] and post mortem brain tissue of autistic individuals [56] were repeatedly reported. Similar finding were also reported for IL-1 [57, 58].

MMPs have also the ability through their cleavage mechanisms and proteolytic processing to regulate chemokines gradient, and therefore, control the influx of leukocytes [59]. Chemokines have been repeatedly reported to be associated with ASD [60]. For example, we reported recently elevated levels of monocyte chemotactic protein-1 (MCP-1) (a CC chemokine encoded on human chromosome 17) in amniotic fluid samples of cases that developed ASD later in life [61]. Interestingly chemokines share overlapping pathways with MMPs through which they contribute to the neuropathology of ASD. The role of some chemokines (such as MCP-1) in neuroinflammation has been well established using the animal model of EAE where a positive correlation between the expression and the degree of inflammation in the CNS was reported [62]. Furthermore, this chemokine can act similarly to MMPs and induce BBB breakdown [63].

Taken together, MMPs can contribute to the etiopathology of ASD through several pathways which are not necessarily mutually exclusive. MMPs can modulate neuroplasticity and neurogenesis and contribute to a hyperplasticity state associated with ASD. Their pathologic proteolytic effects on the BBB can also leave the CNS vulnerable to the systemic circulation in critical developmental periods. Furthermore, MMPs can hinder neurodevelopment through inducing neuroinflammatory state within the CNS and through their proteolytic effects on NFs, cytokines and chemokines.

## Conclusion

MMPs encompass a large family of proteases that share many similarities in their structure, regulation and function. These enzymes play crucial role not only in the normal development of the central nervous system but also in a number of key pathologic processes including inflammation, fibrosis, arthritis and cancer. Contribution of MMPs to the etiopathology of ASD is biologically plausible through direct and indirect pathways that are not necessarily mutually exclusive. This includes their role in neuroinflammation, BBB disruption and their modula-tory enzymatic effects on key biomarkers such as cytokines, chemokines and NFs. While MMPs have been extensively studied in several pathologies, their role in ASD was less extensively examined. In this review we presented the current evidence of MMPs contribution to ASD pathology. Despite the biologic plausibility, further research in this area including examining levels of MMPs in different stages during pregnancy and after birth is needed to identify their pattern in ASD and their associations with other markers such as cytokines and NFs.

## Abbreviations

ADAMs: A disintegrin and metalloproteinases
ADAMTs: A disintegrin and metalloproteinase with thrombospondin motifs
ASD: Autism spectrum disorders
BBB: Blood–brain barrier
BDNF: Brain-derived neurotrophic factor
CNS: Central nervous system
EAE: Experimental allergic encephalomyelitis
ECM: Extracellular matrix
FXS: Fragile x syndrome
LTP: Long-term potentiation
MMPs: Matrix metalloproteinase
MT-MMPs: Matrix metalloproteinase
NFs: Neurotrophic factors
ROS: Reactive oxygen species.
